# Exploiting the Modulation Effects of Epitaxial Vanadium Film in a Quasi-BIC-Based Terahertz Metamaterial

**DOI:** 10.3390/ma18102197

**Published:** 2025-05-10

**Authors:** Chang Lu, Junxiao Liu, Sihong Chen, Junxiong Guo

**Affiliations:** 1 Department of Electronic Communication and Technology, Shenzhen Institute of Information Technology, Shenzhen 518172, China; luchang2010@foxmail.com; 2 School of Electronic Science and Engineering, University of Electronic Science and Technology of China, Chengdu 611731, China; 202121020627@std.uestc.edu.cn; 3 School of Electronic Information and Electrical Engineering, Institute for Advanced Study, Chengdu University, Chengdu 610106, China

**Keywords:** metamaterial, Quasi-BIC, vanadium dioxide, metal–insulator transition

## Abstract

Terahertz (THz) metamaterials based on phase-change materials (PCMs) offer promising approaches to the dynamic modulation of electromagnetic responses. In this study, we design and experimentally demonstrate a tunable THz metamaterial composed of a symmetric split-ring resonator (SRR) pair, with the left halves covered by a 35 nm thick epitaxial vanadium dioxide (VO_2_) film, enabling the simultaneous exploitation of both permittivity- and conductivity-induced modulation mechanisms. During the metal–insulator transition (MIT) of VO_2_, cooperative changes in permittivity and conductivity lead to the excitation, redshift, and eventual disappearance of a quasi-bound state in the continuum (QBIC) resonance. Finite element simulations, using optical parameters of VO_2_ film defined by the Drude–Smith model, predict the evolution of the transmission spectra well. These results indicate that the permittivity change originating from mesoscopic carrier confinement is a non-negligible factor in THz metamaterials hybridized with VO_2_ film and also reveal the potential for developing reconfigurable THz metamaterials based on the dielectric modulation effects of VO_2_ film.

## 1. Introduction

Terahertz (THz) waves, referring to electromagnetic radiation between 0.1 and 10 THz, occupy a distinctive position in the electromagnetic (EM) spectrum between radio and optical frequencies [1,2,3]. They are promising for both next-generation communication technologies [4,5,6,7,8,9], offering advantages including abundant bandwidth, high data rates, and low latency, and sensing and imaging applications [10,11,12,13,14,15,16], enabling nonionizing, nondestructive detection with large penetration depth across various materials. Breakthroughs in THz wave generation, manipulation, reception, and signal processing techniques continue to emerge [17,18,19,20], spanning both photonics-based [21,22,23,24] and all-electronic-based approaches [25,26].

In addition to optics and semiconductor devices, THz metamaterials have attracted significant attention in recent years for their ability to manipulate electromagnetic waves using artificially designed subwavelength structures [27,28,29]. Reconfigurable metamaterials, which combine active components with metamaterial platforms, enable dynamic control under external stimuli [30]. Approaches to achieving reconfigurable EM metamaterials include micro-electro-mechanical system (MEMS) techniques [31,32,33], semiconductor-based designs [34], flexible frameworks [35,36], phase-change materials (PCMs) [37,38,39], and others. Among these, PCMs have been widely studied in the THz regime due to their hysteresis effects, material diversity, and fabrication flexibility. Four types of PCMs are mainly employed at THz frequencies: (1) conductivity transition materials like GST and VO_2_, unitized either as a film-type medium to provide controllable overall transmission or reflectance [40,41,42] or in the form of subwavelength connectors between metallic arms to achieve conducting switching [43,44,45]; (2) ferroelectric materials such as STO, whose permittivity alters in response to thermal stimuli (from 136 °C to −123 °C) and electric fields, enabling the tunability of the dielectric environment when used as a substrate medium [46,47,48,49,50,51]; (3) liquid crystal–hybrid metamaterials, in which liquid crystal films enable controllable absorption and birefringence effects by adjusting molecular alignment under external electric fields [52,53,54,55]; and (4) superconductor materials like YBCO [56,57] and NbN [58], generally patterned into subwavelength resonators, providing tunable resonant strength through a dramatic reduction in ohmic loss below the critical temperature.

Among these PCMs, VO_2_ has attracted considerable attention due to its easily accessible critical temperature (50–72 °C, varies in different kinds of films), significant conductivity changes spanning 0.1 to 1000 S/cm, and strong modulation effects with a small material volume that accompanies the metal–insulator transition (MIT) [30,38,59,60,61,62,63,64,65,66]. However, while previous studies have primarily focused on its conductivity transition, the accompanying permittivity changes, arising from mesoscopic carrier confinement, remain largely unexplored [67,68]. These permittivity changes, which behave as time delay to THz waves, are difficult to detect exactly in bare VO_2_ film. However, they can significantly influence the modulation effect in VO_2_ film–hybrid THz metamaterials, due to the enhanced light–matter interaction. Several works have revealed permittivity-based modulation effects in VO_2_–hybrid THz metamaterials, but deeper investigations into this phenomenon are still lacking [69,70]. Furthermore, these permittivity effects provide opportunities for developing dielectric-type reconfigurable metamaterials based on VO_2_ film, which offer simpler triggering conditions compared to other PCMs that enable permittivity tunability. Thus, exploiting dielectric-induced modulation effects in VO_2_-based THz metamaterials holds both fundamental physical significance and practical application value.

In this study, we propose a THz metamaterial based on the quasi-bound state in the continuum (QBIC) mechanism [71,72,73,74] to experimentally demonstrate the permittivity-induced modulation effect in 35 nm epitaxial VO_2_ film. The unit cell of this metamaterial consists of two similar split-ring resonators (SRRs), with one of them being covered by a VO_2_ film. This type of QBIC metamaterial, based on the interference of two distinct resonances, is known for its high sensitivity to dielectric changes in the half-covered film [71]. Finite element simulations show that the emergence of QBIC resonance is dominated by the permittivity change in the VO_2_ film, while the conductivity increase mainly influences the Q-factor. Therefore, the observed QBIC resonances in the experimental results—characterized by excitation, redshift, and eventual disappearance during the MIT—reveal the cooperative effects of permittivity- and conductivity-induced asymmetry across the SRR pair. Furthermore, the Drude–Smith model, relating the optical properties of VO_2_ to temperature, was used in simulations and turned out to predict the transmission spectrum evolution well. In the Section 3.6, we compare the triggering conditions and optical properties of various PCMs capable of inducing permittivity changes in the THz regime, highlighting the advantages of VO_2_ films in terms of their broad permittivity range (from 80 to 1600) and near-room-temperature phase transition.

## 2. Materials and Methods

**Figure 1:**
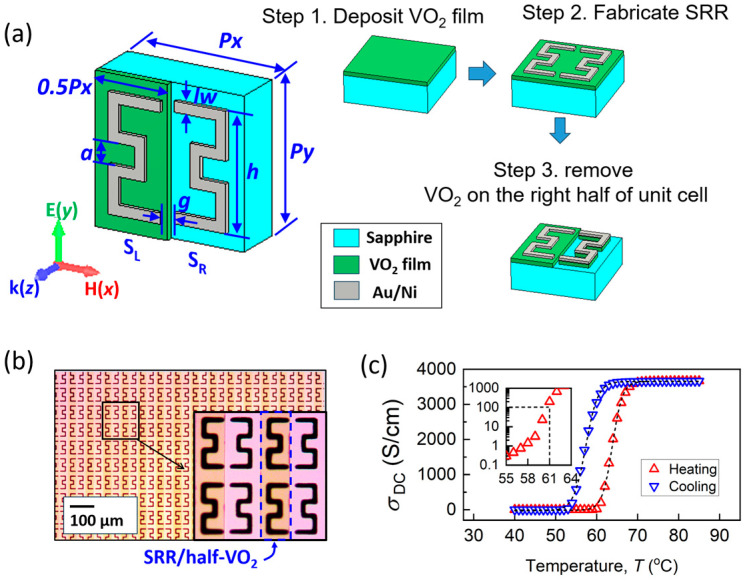
Fabrication and characteristics of QBIC metamaterial. (**a**) Metamaterial unit cell consists of split-ring resonators (Au/Ni, 200/10 nm), half-covered VO_2_ film (35 nm), and sapphire substrate (500 μm). Fabrication steps are schematically shown in right panel. Structural parameters are as follows: Px = Py = 70 μm; h = 55 μm; a = 16 μm; lw = 5 μm; and g = 5 μm. Lateral length of VO_2_ strip is 0.5Px. (**b**) Microscopic image of metamaterial, with inset showing enlarged unit cells. (**c**) Temperature-dependent DC conductivity of VO_2_ film, with inset showing critical temperature (Tc) for conductivity transition at 61 °C.

Figure 1a schematically shows the design of the reconfigurable metamaterial composed of SRR/half-VO_2_ arrays. A single unit cell consists of a pair of SRRs (Au/Ni, 200/10 nm), an epitaxial VO_2_ thin film (35 nm, half-covered), and a sapphire substrate (500 μm). The structural parameters are as follows: Px = Py = 70 μm; h = 55 μm; a = 16 μm; lw = 5 μm; and g = 5 μm. To disrupt the symmetry, a VO_2_ strip with a lateral width of 0.5Px covers the left half of the unit cell, denoted as SRR/half-VO_2_. While SRR structures are well understood for their LC-type resonance, the coupling between SRR/VO_2_ and SRR within the same unit cell enables symmetry breaking triggered by the MIT of the VO_2_ film. Throughout the manuscript, finite element simulations are conducted by CST Microwave Studio 2016.

The fabrication process, shown in the right panel of Figure 1a, consists of three steps: (1) depositing a 35 nm epitaxial VO_2_ film using the polymer-assisted deposition method; (2) lithographing the mask for the SRR pattern (UV contact exposure machine: RE-2000/35, Institute of Microelectronics, Chinese Academy of Sciences, Beijing, China) and depositing the SRR (Au/Ni, 200/10 nm) by magnetron sputtering (BMS560B, BEIJING KEVIC, Beijing, China); and (3) lithographing the mask for the VO_2_ strips (AZ6112) and removing the uncovered VO_2_ film through dry etching (DRIE). Dry etching is carried out using a gas mixture of 50 sccm SF_6_ and 5 sccm O_2_ at 100 W power for 60 s, achieving an etching depth of 35 nm. Due to the high selectivity of this gas mixture between VO_2_ and Au, dry etching is performed without damaging the gold film. A microscopic photograph of the as-prepared metamaterial is shown in Figure 1b, with an inset showing the enlarged unit cells, highlighting the VO_2_ film covering the left half of the SRR pair.

The epitaxial VO_2_ films were deposited onto an m-cut sapphire substrate using the polymer-assisted deposition method, with the epitaxial relationship (−402) VO_2_ || Al_2_O_3_ (10-10), as confirmed by the X-ray diffraction (XRD) patterns shown in Figure S1 (Supporting Information). Sapphire was selected as the substrate due to its excellent lattice matching with VO_2_. The monoclinic-to-tetragonal phase transition of VO_2_ aligns well with the hexagonal lattice of sapphire, which is critical for achieving high-quality epitaxial growth and ensuring a well-defined and repeatable phase transition [66]. The MIT quality of 35 nm VO_2_ film is demonstrated by the DC conductivity measurements presented in Figure 1c, where a sharp change of over four orders of magnitude, with a hysteresis loop width of ~6.5 °C, is observed. The Tc of the conductivity transition, identified from the transition threshold (the inset of Figure 1c), is 61 °C during the heating process.

## 3. Results

### 3.1. Mechanisms for QBIC

To better analyze the spectral change in the QBIC metamaterial induced by the MIT, we first analyze the permittivity- and conductivity-induced modulation effects separately and then present a cooperative study in the following section.

**Figure 2:**
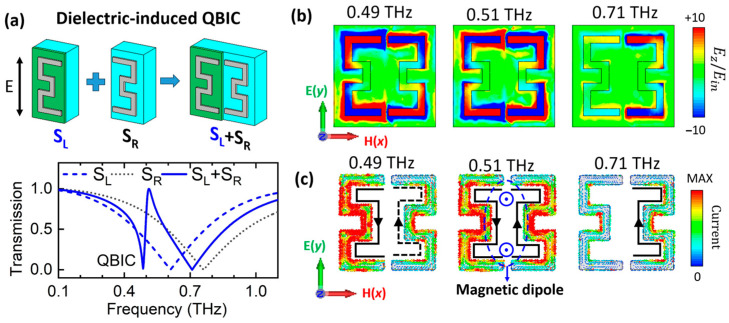
Coupling mechanism for permittivity-induced QBIC for $n_{d}=30$, $\sigma_{1}=0$. (**a**) Schematics and calculated spectra of three metamaterial configurations: S_L_ for SRR fully covered by VO_2_ (blue dashed line), S_R_ for SRR only (balck dotted line), and S_L_ + S_R_ (blue solid line) for combined S_L_ and S_R_ (QBIC metamaterial, spectra shown in solid line). (**b**) Electric field Ez (normalized to input electric field) and (**c**) current distributions of QBIC metamaterial at 0.49 THz (QBIC dip), 0.51 THz (transmission peak), and 0.71 THz (LC resonance). The black arrows indicate the currents directions.

The QBIC mechanism in this metamaterial arises from the asymmetry between the left and right SRRs, representing the interference of two different resonances within the same channel [71]. Therefore, it is crucial to compare the resonance between each half to evaluate the asymmetry degree. Thus, we developed three sets of metamaterials, as illustrated in Figure 2a. The first array consists of the left half of the unit cell (S_L_), with the SRR fully covered by the VO_2_ film; the second consists of the right half of the unit cell (S_R_), with only the SRR; and the third (the QBIC metamaterial) consists of both the left and right halves, i.e., the SRR/half-VO_2_ array. In the calculations, the Al_2_O_3_ substrate was treated as a lossless dielectric material with permittivity ~11.1, and the SRRs were modeled as perfect electric conductors. All the spectra calculated in this work are based on the finite element method with a periodic unit cell boundary.

The coupling mechanism in a permittivity-induced QBIC is illustrated in Figure 2a. When the VO_2_ film has a high refractive index ($n_{d}=30$) and zero electrical conductivity ($\sigma_{1}=0$), the spectra of the S_L_ and S_R_ arrays exhibit broad resonances, which is assigned to LC-type excitation. The resonance frequency of S_L_ (blue dotted line) is lower than that of S_R_ (blue dashed line) due to the high permittivity of the VO_2_ film. The difference in resonance frequency between S_L_ and S_R_ results in asymmetric excitation when coupled within a single unit cell, producing a narrow Fano-type resonance, as shown in Figure 2a (blue solid line).

To illustrate this explanation, the electric field (z-polarized, shown in Figure 2b) and current (Figure 2c) distributions for the QBIC metamaterial are calculated at three representative frequencies. As shown in Figure 2c, a pronounced head-to-tail closed current distribution over the SRR pair is observed at 0.51 THz, generating out-of-plane magnetic dipoles (MDs). The MDs trap energy in local fields and induce destructive interference, producing an enhanced transmission peak at 0.51 THz. At 0.49 THz, the currents excited on S_L_ are slightly stronger than those on S_R_, leading to approximately destructive interference and a transmission dip. At 0.71 THz, currents focus on S_R_, giving rise to broad electric dipole resonance. It is evident that the field enhancement at QBIC resonance is much stronger than that at dipole resonance, implying a stronger light–matter interaction and greater material sensitivity of the QBIC resonance compared to the LC resonance.

**Figure 3:**
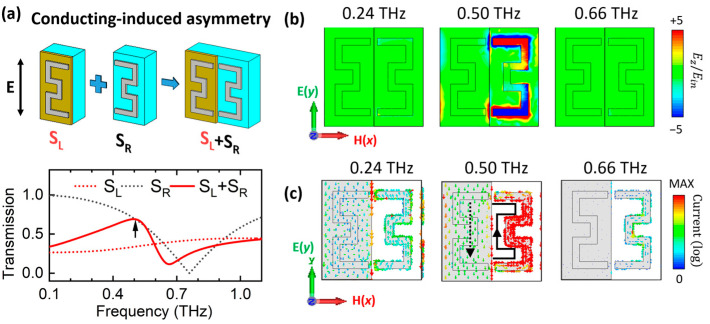
Coupling mechanism for conducting-induced transmission peak for $n_{d}=3$, $\sigma_{1}=3500$ S/cm. (**a**) Schematics and calculated spectra of three metamaterial configurations: S_L_ for SRR fully covered by VO_2_ (red dotted line), S_R_ for SRR only (black dotted line), and S_L_ + S_R_ for combined S_L_ and S_R_ (red solid line). (**b**) Electric field Ez (normalized to input electric field) and (**c**) current distributions (in log scale) at 0.24 THz, 0.50 THz (transmission peak), and 0.66 THz (LC resonance). The black arrows indicate the currents directions.

In the conducting state of the half-VO_2_ ($n_{d}=3$, $\sigma_{1}=3500$ S/cm), conducting electrons in the VO_2_ film allow current to leak between the capacitive gap and screen the LC resonance on S_L_, resulting in an overall decrease in the transmission spectra of the S_L_ array (red dotted line in Figure 3a). Meanwhile, S_R_ maintains the broad LC-type resonance (black dotted line). The coupling between these two leads to a Fano lineshape across the transmission spectrum, with an enhanced transmission peak at 0.50 THz (red solid line). The QBIC dip disappears due to screening by conducting electrons in the VO_2_ film. The enhanced transmission peak at 0.50 THz is associated with weak currents on the VO_2_ film, generating head-to-tail closed current distributions (Figure 3c) and out-of-plane magnetic dipoles, leading to field enhancement at 0.50 THz, as shown in the electric field map in Figure 3b.

### 3.2. Cooperative Permittivity–Conductivity-Induced Modulation Effects

**Figure 4:**
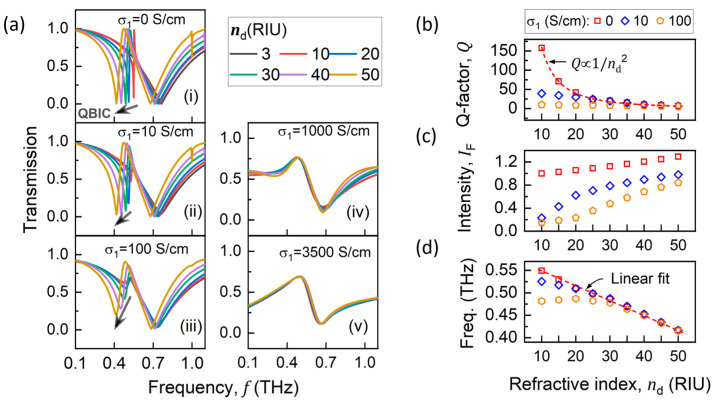
(**a**) Transmittance spectra showing QBIC resonances under comprehensive effects of $\sigma_{1}$ and $n_{d}$. (**b**) Q-factor, (**c**) normalized intensity, and (**d**) resonance frequency shift in QBIC as functions of $\sigma_{1}$ and $n_{d}$. Intensity is normalized to QBIC intensity value at $n_{d}=10,\;\sigma_{1}=0.$.

The MIT of the VO_2_ film involves simultaneous changes in both its permittivity and conductivity properties. To investigate this cooperative effect, transmission spectra were calculated for the refractive index ($n_{d}$) ranging from 3 to 50 and a real-component conductivity ($\sigma_{1}$) of 0, 10, 100, 1000, and 3500 S/cm, as shown in Figure 4a. In order to quantify and compare the QBIC resonances in Figure 4a(i–iii), we fitted the transmittance spectra as a Fano resonance (the QBIC resonance) on a Lorentzian background (the LC resonance). Details and examples for the fitting process are provided in Figure S2a,b, Supporting Information. The corresponding resonance properties of QBIC, including Q-factor (Figure 4b), normalized intensity (Figure 4c), and frequency shift in the resonance dip (Figure 4d), are summarized in Figure 4b–d as a function of $n_{d}$.

The case with σ_1_ = 0, shown in Figure 4a(i), corresponds to a lossless condition and reflects a pure permittivity-induced symmetry-breaking mechanism. In this case, the Q-factor of QBIC resonance is inversely proportional to ${n_{d}}^{2}$ (red dotted line in Figure 4b), the intensity increases with $n_{d}$, and the resonance frequency shift shows a linear relationship with $n_{d}$, in agreement with the theoretical model proposed by Thomas et al. [71].

In the low-conductivity cases ($\sigma_{1}=10$ and 100 S/cm) shown in Figure 4a(ii,iii), Q-factors and resonance intensities are lower compared to those at $\sigma_{1}=0$. However, when the VO_2_ film is in a highly conducting state ($\sigma_{1}$ ≥ 1000 S/cm), the QBIC resonance disappears (Figure 4a(iv,v)), and the spectra become nearly unaffected by $n_{d}$. Extended simulations at fixed $n_{d}$ and $0<\sigma_{1}<10^{7}$ S/cm (shown in Figure S2c, Supporting Information) further demonstrate this conclusion.

In conclusion, the emergence of QBIC resonance is dominated by the permittivity change in the VO_2_ film, while the conductivity change mainly influences the resonance intensity and quality factor. To evaluate the influence of $n_{d}$ and $\sigma_{1}$ on resonance frequency, we defined the change rate of resonant frequency with respect to $n_{d}$ as follows: sensitivity= $∆f/∆n_{d}$, where ∆f is the frequency shift, and ${∆n}_{d}$ represents the refractive index change in VO_2_ film. The sensitivity obtained from the linear fit to data shown in Figure 4d is −3.2 GHz/RIU, −2.76 GHz/RIU, and −1.72 GHz/RIU for $\sigma_{1}$ values of 0, 10, and 100 S/cm, respectively. This indicates a decreased value in the dielectric sensitivity of QBIC resonance as electrical conductivity increases.

### 3.3. THz Properties of VO_2_ Film

To better understand the modulation effect in this metamaterial enabled by the MIT, it is crucial to establish a reliable model for the THz properties of the VO_2_ film. It is generally recognized that VO_2_ undergoes an inhomogeneous MIT due to its first-order phase transition nature, during which metallic (rutile) and insulating (monoclinic) phases coexist and compete. Pronounced carrier confinement effects arise during this process, influencing both permittivity and electrical conductivity. These effects can be described using the Drude–Smith formula [75]:

(1) $$\tilde{\sigma }(\omega )=\frac{Ne^{2}\tau /m*}{1-i\omega \tau }(1+\frac{c_{1}}{1-i\omega \tau })$$

where $\sigma =\sigma_{1}+i\sigma_{2}$ is the complex electrical conductivity, N is the carrier density, e is the elementary charge, m∗ is the effective mass of charge carriers, τ is the scattering time, and $c_{1}$ is the confinement factor. Specifically, we set the constants as $m*=23m_{e}$ and τ=20 fs and define the temperature-dependent factors as N ranging from 0 to 5 × 10^22^ cm^−3^ and $c_{1}$ ranging from −1 to −0.75, based on our previous research about the THz properties of epitaxial VO_2_ film [76]. More details about this model are provided in Section S3, Supporting Information. In recent years, researchers have attempted to provide a physical explanation for the evolution of the $c_{1}$ parameter within the framework of Monte Carlo calculations, where $c_{1}$ is related to the relative size of metallic domains compared to the carrier diffusion length [77]. In this framework, the variation in $c_{1}$ from −1 to −0.75 with increasing temperature is assigned to the growth of metallic domains—from small, isolated clusters at the onset of the MIT to larger connected domains as the phase transition progresses. This behavior is consistent with microscopic observations of phase evolution in epitaxial VO_2_ films [78,79,80,81]. Raman spectroscopy analysis, provided in Section S3 of the Supporting Information, confirms that the observed modulation effects are associated with the general metal (monoclinic phase)-to-insulator (rutile phase) transition of VO_2_ film.

**Figure 5:**
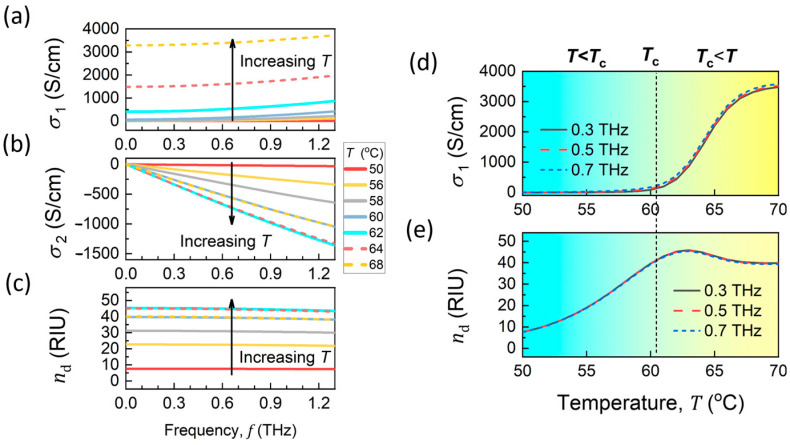
(**a**) Real and (**b**) imaginary components of complex conductivity ($\sigma_{1}$ and $\sigma_{2}$) at several representative temperatures in heating process. (**c**) Refractive index ($n_{d}$) calculated according to $\sigma_{2}$. (**d**,**e**) Temperature-dependent $\sigma_{1}$ and $n_{d}$ at 0.3, 0.5, and 0.7 THz for VO_2_.

The results of the Drude–Smith model are shown in Figure 5a–c for several representative temperatures. The positive $\sigma_{1}$ in Figure 5a, which exhibits a slight slope with respect to frequency, and $\sigma_{2}$, which has negative values, as shown in Figure 5b, are characteristic of the carrier confinement effect in nanostructured VO_2_ film, consistent with the experimental results [68,76]. The refractive index $n_{d}$ (Figure 5c) is calculated according to ${n_{d}}^{2}=\varepsilon_{\infty }-\sigma_{2}/\omega \varepsilon_{0}$, where $\varepsilon_{\infty }$ is set to 9, and $\varepsilon_{0}$ is the free space dielectric constant. It is shown that the carrier confinement effect modeled by Equation (1) does not lead to significant dispersion in $\sigma_{1}$ and $n_{d}$ within the frequency range of 0.3 to 0.7 THz (corresponding to the QBIC and LC resonances). Figure 5d,e present $\sigma_{1}$ and $n_{d}$ as functions of temperature at 0.3, 0.5, and 0.7 THz, where their traces nearly overlap. Therefore, $\sigma_{1}$ and $n_{d}$ are treated as frequency-independent values in this work.

The Drude–Smith model suggests the existence of a “dielectric window” at temperatures below the critical temperature Tc (61 °C), where the VO_2_ film undergoes a dielectric change, while its conductivity remains at a low value ($\sigma_{1}<$ 100 S/cm). This “dielectric window” is supported by experimental observations and is attributed to fully confined electrons below Tc [76,82]. At temperatures around Tc (60–62 °C), the value of $n_{d}$ stabilizes, while electrical conductivity rapidly increases from about 10 to 500 S/cm. At temperatures well above Tc, $\sigma_{1}$ continues to increase and reaches the maximum, approximately 3500 S/cm at 70 °C.

**Figure 6:**
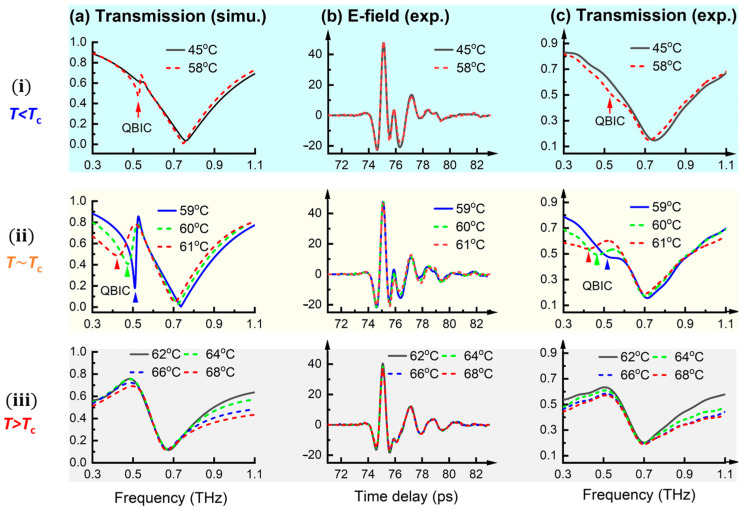
Experimental and simulated spectra as functions of temperature. (**a**) Temperature-dependent spectra simulated using VO_2_ properties modeled by Drude–Smith function. (**b**) Measured time-domain signals and (**c**) transmission spectra of QBIC metamaterial in heating process.

The simulations of the transmission spectra, using the Drude–Smith model for the THz properties of VO_2_, are shown in Figure 6a(i–iii). For better clarification, the MIT process depicted in Figure 6 is divided into three stages: (i) T < Tc (40–58 °C), representing the early stage of the MIT; (ii) T ≈ Tc (59–61 °C), corresponding to the transition region near Tc; and (iii) T > Tc (62–70 °C), representing the post-transition stage. At stage (i), the modulation effects induced by the MIT appear as permittivity-induced asymmetry across the SRR pair, leading to the emergence of a sharp QBIC dip (indicated by the arrow) around 0.52 THz, as shown in Figure 6b(i). With increasing temperature at stage (ii), the QBIC resonance redshifts and its spectral features become less pronounced, as shown in Figure 6b(ii). Meanwhile, the simulation results also indicate a decreasing Q-factor as T increases from 59 to 61 °C, because σ₁ exceeds 100 S/cm at this stage, degrading the resonance quality of the QBIC mode. Thus, the modulation effect at this stage is characterized by a cooperative permittivity- and conductivity-induced asymmetry. At stage (iii), when T > Tc (Figure 6a(iii)), the QBIC resonance disappears, and the transmission spectra exhibit an overall Fano-like asymmetry. In this stage, conductivity-induced asymmetry dominates the transmission behavior as σ_1_ exceeds 1000 S/cm.

### 3.4. Experimental Spectra

The transmission spectra of the QBIC metamaterial were measured using fiber-coupled THz time-domain spectroscopy (THz-TDS), with the sample placed on a high-resolution temperature controller capable of ~0.01 °C precision. Detailed experimental setup information, including the THz-TDS schematic, practical photographs of the devices, and the equipment bandwidth, is provided in Section S5 of the Supporting Information. The time-domain signal, recorded with a sampling rate of 20 fs, 1000 repetition times, and a time window length of 12 ps (with Fabry–Pérot echoes within the substrate removed), is shown in Figure 6b for several representative temperatures during the heating process (transmission spectra for the cooling process are provided in Figure S6a, Supporting Information). The corresponding Fourier-transformed, frequency-dependent spectra, normalized to a bare sapphire substrate (showing a ~20% decrease in transmission amplitude due to substrate loss), are shown in Figure 6c.

As seen in Figure 6b(i,ii), the modulation effects induced by the MIT appear as subsidiary waves following the main pulse in the time-domain signal. This behavior reflects a shift in resonance mode, as shown in Figure 6c(i,ii). However, different from the sharp QBIC resonance predicted in simulations, the measured transmission spectra at 58–59 °C only exhibit an abrupt change at the spectral position of the predicted QBIC mode. This QBIC mode subsequently evolves into a well-defined resonance and redshifts to 0.44–0.42 THz as the temperature increases to 60–61 °C. At temperatures above 61 °C, the QBIC resonance disappears, and an enhanced transmission peak appears at 0.50 THz, attributed to conductivity-induced asymmetry across the SRR pair.

The simulations predict the spectral profile well, including the position of the QBIC mode (denoted by arrows). This indicates that the Drude–Smith model accurately predicts the permittivity and conductivity of the VO_2_ film. Specifically, the QBIC resonance in the experimental transmission spectra at 60 °C exhibits a Q-factor of 3.4, which is much lower than the predicted Q-factor of 8.5 from the simulation spectra. Similarly, at 61 °C, the Q-factors of the QBIC resonance in the experimental and simulation spectra are 4.2 and 7.0, respectively. A primary reason for this discrepancy is the limited time signal length in the experimental results, as the QBIC resonance is a long-duration oscillation. As shown in Figure 6b, the time signal after 83 fs is removed due to interference with the Fabry–Pérot echoes within the 500 μm sapphire substrate. While increasing the substrate thickness could help acquire a longer time signal before the Fabry–Pérot pulses arrive, it would also significantly increase substrate loss. Additional experimental factors, such as unexpected energy loss, limited signal-to-noise ratio, and geometric defects in the unit cells and array, also contribute to this discrepancy. A promising solution to addressing the substrate limitation involves peeling off the VO_2_ film from the dielectric substrate to create a free-standing metasurface, which is an advanced fabrication technique reported by other researchers [83,84].

### 3.5. Frequency-Dependent Modulation Effects

**Figure 7:**
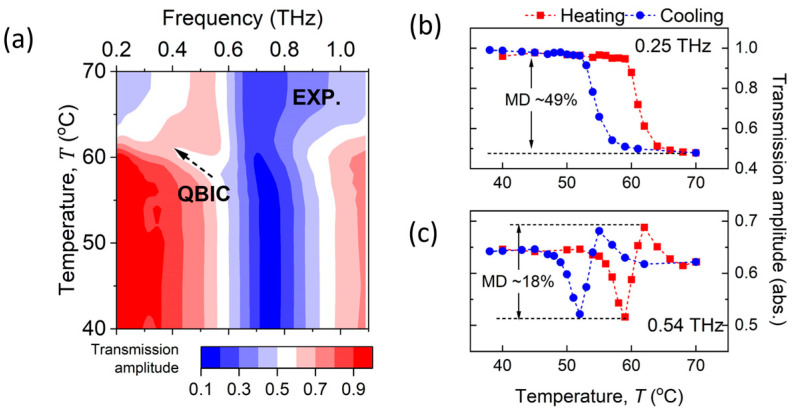
(**a**) Transmission amplitude in heating process as function of temperature and frequency, with QBIC frequency traced by black arrow. (**b**,**c**) Temperature-dependent transmission amplitude at 0.25 THz and 0.54 THz, showing hysteresis and reversible modulation effects in heating and cooling cycles.

As shown in Figure 7a, the transmission amplitude during the heating process is plotted as a function of temperature and frequency, with the QBIC resonance traced by a black arrow. Figure 7b,c illustrate the modulation effects in two representative cases: one at 0.25 THz, which is well below the QBIC trace, and another at 0.54 THz, which intersects the QBIC trace. It can be observed that the transmission amplitude at 0.25 THz continuously decreases in the heating process and only recovers in the cooling process, exhibiting a thermal hysteresis loop with a width of approximately 7 °C. In contrast, the transmission at 0.54 THz shows reversible behavior within a single heating process, as a result of cooperative permittivity–conductivity-induced modulation. It first decreases at 50–59 °C, attributed to the emergence of the QBIC resonance; subsequently increases at 61–63 °C, due to the redshifts in the QBIC resonance; and then decreases again at 62–70 °C, as the QBIC resonance disappears. Thus, the modulation effects at 0.54 THz exhibit a tri-stage response compared to the monotonous behavior at 0.25 THz, leading to a smaller net modulation depth but higher repetition rate at 0.54 THz.

**Figure 8:**
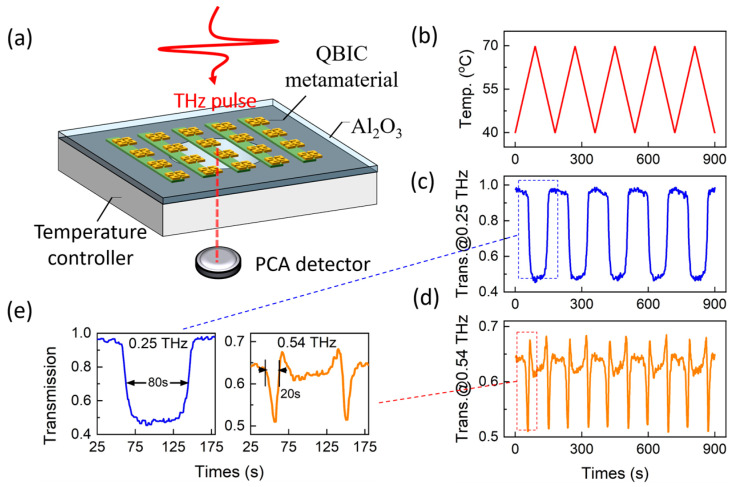
(**a**) Experimental setup for dynamically modulating transmission signal. (**b**) Temperature serials as function of time. (**c**,**d**) Time-traced transmission at 0.25 THz and 0.54 THz showing rectangular wave and sharp pulse modulation. (**e**) Switching cycle with recovery times for both frequencies.

The frequency-dependent modulation effect implies the potential for different types of signals modulated by precise temperature controlling. This is demonstrated experimentally by using the setup shown in Figure 8a, where the QBIC metamaterial is placed on a temperature controller operating at a fixed heating and cooling rate, as shown in Figure 8b (20 °C/min with ~0.1 °C precision, using resistance wire for heating and liquid nitrogen for cooling). Meanwhile, the transmitted time-domain signal is continuously monitored by a PCA detector. The corresponding time-traced transmission, normalized to a bare sapphire substrate, is shown in Figure 8c for 0.25 THz, where a rectangular wave is modulated by the temperature controller. In contrast, the time-traced transmission at 0.54 THz in Figure 8d exhibits sharp pulses with narrower time widths and a doubled repetition rate compared to the signal at 0.25 THz. Figure 8e shows one complete switching cycle, revealing that the recovery and falling times are 80 s and 13 s for 0.25 THz, respectively, and 20 s and 7 s for 0.54 THz. Thermal stability within 45 cycles of heating and cooling is presented in the Supporting Information, Section S6.

### 3.6. PCMs with Permittivity Tunability

Permittivity-induced modulation effects at THz frequencies are observed not only in VO_2_ films but also in several other PCMs. We summarize the types, mechanisms, structures, triggering conditions, and permittivity changes in these materials in Table 1.

For ferroelectric materials such as BTO and STO, the permittivity change is attributed to crystal lattice reorientation under external electric fields or temperature control. A related study by Ranjan Singh et al. reported on metal resonators fabricated on a bulk STO substrate, revealing a pronounced modulation effect on the resonance frequency, shifting from 0.225 to 0.125 THz as the temperature decreased from 136 to −123 °C [48]. Other works utilizing BTO film [51] or STO nanopowders [46] reported less pronounced permittivity modulation due to the limited volume of the PCM. Liquid crystal materials, based on the birefringence effects of realigned molecules, exhibit small changes in permittivity (less than 0.5, as reported in references [85,86]). In the case of VO_2_, the permittivity change originates from the mesoscopic carrier confinement effect during the MIT. As summarized in Table 1, VO_2_ films exhibit a larger scale of permittivity change and a critical temperature much closer to room temperature compared with other PCMs. However, previous researchers found that this effect is detected in 35 nm film but weakens when the film thickness is increased to 85 nm [76,82]. Another study by Wanlin Liang et al. observed a similar permittivity-induced modulation effect in metamaterials hybridized with a 10 nm VO_2_ film [69]. This suggests that the permittivity changes in VO_2_ films are influenced by film morphology and are not as stable as the intrinsic conductivity change induced by the phase transition. However, it also highlights the potential for improving the permittivity effect through fabrication techniques, offering promising opportunities for advancing dielectric-type reconfigurable metamaterials based on VO_2_ films.

## 4. Conclusions

In conclusion, this study presents and experimentally demonstrates a metamaterial design where a symmetric SRR pair is half-covered by a 35 nm thick epitaxial VO_2_ film, enabling a cooperative permittivity- and conductivity-induced modulation effect during the MIT. At the onset of the MIT (58 °C), an abrupt change in the transmission spectra is observed at 0.53 THz, attributed to the permittivity-induced QBIC mode. This mode evolves into a well-defined QBIC resonance and redshifts to 0.44–0.42 THz as the temperature increases to 60–61 °C. Above 61 °C, the QBIC resonance disappears, replaced by an enhanced transmission peak at 0.50 THz, due to conductivity-induced asymmetry across the SRR pair. Finite element simulations, using the Drude–Smith function modeled permittivity and conductivity parameters of the VO_2_ film, predict the spectral position of the QBIC mode accurately. Multistate modulation, as a function of temperature, is demonstrated at 0.25 THz and 0.54 THz, revealing pure conductivity-induced modulation and cooperative permittivity–conductivity-induced modulation, respectively. These findings not only enhance the understanding of the THz properties of VO_2_ but also highlight the potential for dielectric-type reconfigurable metamaterials based on epitaxial VO_2_ films.

## Figures and Tables

**Table 1:** Summary of PCM–hybrid THz metamaterials with permittivity tunability.

Material	Phase Transition Mechanism	Metamaterial Structure	Triggering Condition	Permittivity Change in PCM	Published Year
BTO	Ferroelectric	Metal resonator–BTO film	Electric field (0–33 kV/cm)	253 to 185	2014 [51]
STO	Ferroelectric	Metal resonator–STO bulk	Temperature (136 to −123 °C)	219 to 829	2011 [48]
		STO/AuNP/PDMS (all-dielectric metamaterial)	Continuous laser (2.5 W)	10 to 12 * (composite)	2023 [46]
Liquid Crystal (LC)	Adjusting molecular alignment	Metal resonator–LC	Electric field (0–7 kV/m)	2.62 to 2.89	2017 [85]
		Metal resonator–LC	Temperature (25 to 50 °C)	3.6 to 2.9	2018 [86]
VO_2_	MIT	ZrO_2_/VO_2_/PDMS (all-dielectric metamaterial)	Temperature (25 to 100 °C)	5.3 to 10.2 * (composite)	2023 [70]
		Metal resonator–VO_2_ film	Heating circuits (0~0.7 A)	-	2023 [69]
		Metal resonator–VO_2_ film	Temperature (40 to 61 °C)	80 to 1600	This work

* The permittivity changes in all-dielectric metamaterials are calculated based on composite media composed of PDMS (polydimethylsiloxane) and PCM nanopowders.

## Data Availability

The original contributions presented in this study are included in the article/Supplementary Material. Further inquiries can be directed to the corresponding authors.

## Supplementary Materials

The following supporting information can be downloaded at https://www.mdpi.com/article/10.3390/ma18102197/s1, Film characterizations including cross-sectional SEM figures and XRD patterns. Phase diagram of VO_2_ film by in-situ Raman spectroscopy. THz properties of VO_2_ film found by Drude–Smith model. Lorentz and Fano fitting examples. Detailed experiment setup. Experimental transmission spectra in the cooling process, and cycling stability.
