# Supplementary material for: Exploiting the Modulation Effects of Epitaxial Vanadium Film in a Quasi-BIC-Based Terahertz Metamaterial
Source: Materials (Basel). 2025 May 10;18(10):2197. doi: 10.3390/ma18102197 (PMC12112876; doi:10.3390/ma18102197)
Supplement: Supplementary file 1 [file materials-18-02197-s001.zip › materials-3565134-supplementary.pdf]

Supporting Information for

"Exploiting the modulation effect of epitaxial vanadium film in a quasi-BIC based terahertz metamaterial"

Chang Lu [1], Junxiao Liu [2], Sihong Chen [2], and Junxiong Guo [3*]*

[1]*Department of Electronic Communication and Technology,*

Shenzhen Institute of Information Technology,

Shenzhen 518029, China

[2]*School of Electronic Science and Engineering,*

University of Electronic Science and Technology of China,

Chengdu 611731, China

[3]*School of Electronic Information and Electrical Engineering,*

Institute for Advanced Study, Chengdu University,

Chengdu 610106, China

*****Correspondence: sihongchen@uestc.edu.cn, guojunxiong@cdu.edu.cn

Section S1. Film characterization

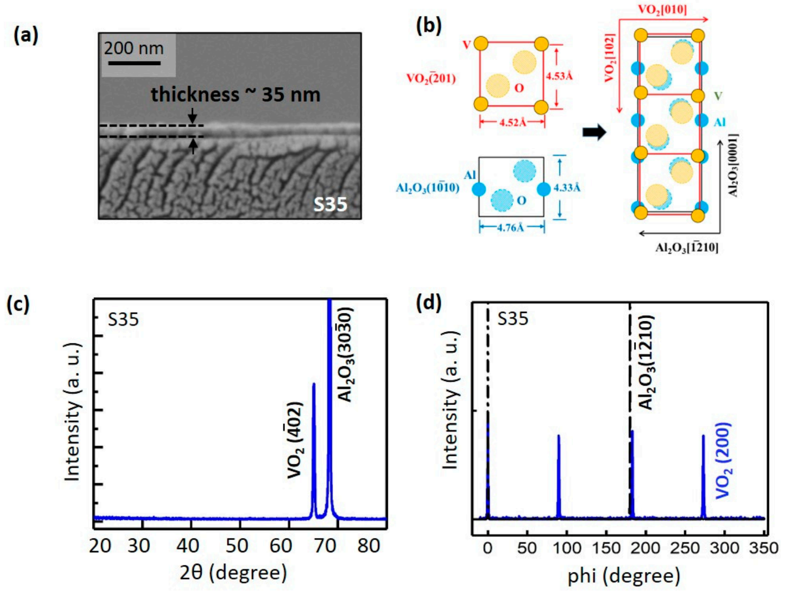

Figure S1. (a) displays scanning electron microscope (SEM) images used to determine the thickness of VO_2 film, which were found to be 35 nm.

Figure S1(b) illustrates the epitaxial relationship between the *m*-sapphire substrate and VO_2 film, which can be summarized as $VO_2(\bar{4}02)$ || $(10\bar{1}0)$ Al_2O_3 in the growth direction and VO_2[102]/[010] || Al_2O_3[0001] in the growth plane. Figure S1(c) shows the room temperature XRD pattern of 35-nm VO_2 film in the $\theta - 2\theta$ scan. The intense $(\bar{4}02)$ reflection at around $2\theta = 65°$ from the *M*1-VO_2 phase confirms the high-quality single-phase VO_2 film. Furthermore, Φ-scan patterns for VO_2(200) diffraction ($2\theta = 37.1^o, \chi = 32.6^o$, blue lines in Figure S1(d), and $(1\bar{2}10)$ Al_2O_3 diffraction ($2\theta = 37.8^o, \chi = 30^o$, black lines) indicate the growth relationship VO_2[102]/[010] || Al_2O_3[0001] in the substrate plane.

Section S2. Lorentz and Fano Fitting Examples & external simulations

The transmittance spectra $T(f) = |t(f)|^2$ is fitted as a Fano resonance T_F (the QBIC resonance) on a Lorentzian background T_d (the dipole resonance):

$$T(f) = T_d + T_F \qquad \text{(S2.1)}$$

$$T_d = T_0 - I_d \frac{(\gamma_d/2)^2}{(f-f_{d0})^2+(\gamma_d/2)^2} \qquad \text{(S2.1a)}$$

$$T_F = -I_F \frac{(W+q)^2}{(1+q)^2(1+W^2)}, W = \frac{f-f_{F0}}{\gamma_F/2} \qquad \text{(S2.1b)}$$

where the subscripts d and F correspond to dipole and Fano respectively; f_0, γ and I are frequency, FWHM and normalized intensity of the corresponding resonance, T_0 is the baseline shift of the whole spectrum, and q is the asymmetry parameter that determines the QBIC resonance profile. The Q-factor in this case is calculated as Q=f_{F0}/γ_F. The fitting process for the simulation results is presented in Figure S2 for two representative cases: (a) n_d= 10, σ_1 = 10 S/cm and (b) n_d = 30, σ_1= 100 S/cm. These well-fitting benefits the analysis of resonant properties of SRR/half-VO_2 spectra.

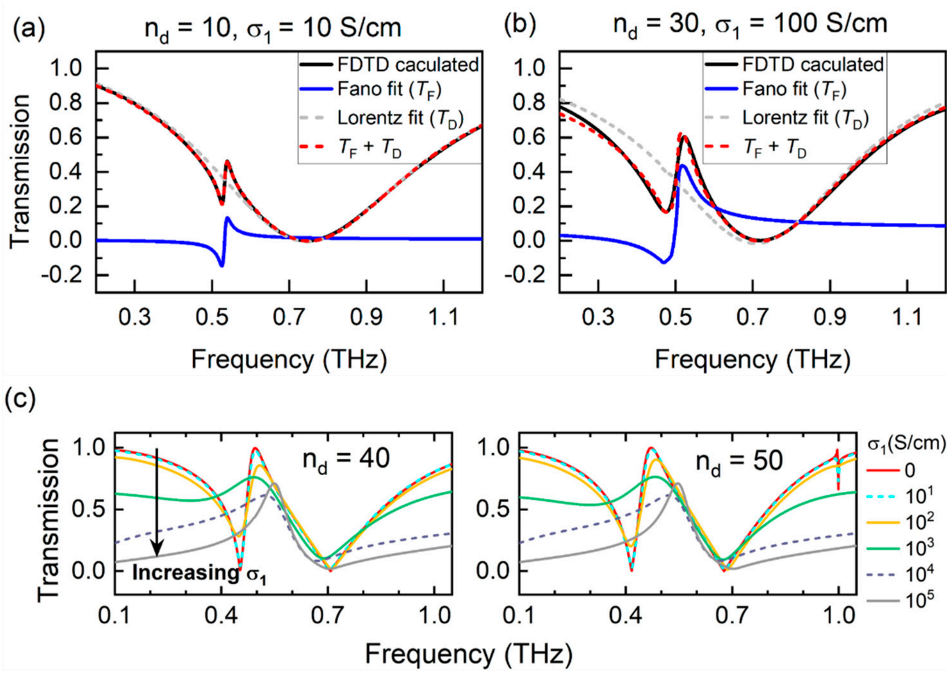

Figure S2. Representative fitting examples of transmittance spectra using Fano resonance function and Lorentzian background. (a, b) Fitting results for simulated spectra with (a) n_d = 10 and σ_1 = 0 S/cm, (b) n_d = 30 and σ_1 = 100 S/cm. (c) Transmission spectra with n_d fixed at 40, 50 and σ_1 changes from 0 to 10^5 S/cm.

Section S3. Phase diagram of VO_2 film by in-situ Raman spectroscopy

To investigate whether microstructural changes occur during the thermal process, we provide supplementary Raman spectroscopy analysis based on our previously published work (Lu, C., et al., *J. Appl. Phys.*, 135, 2024). Figure S3 shows the temperature-dependent Raman spectra of the epitaxial VO_2 film during the metal–insulator transition (MIT). As shown in Figure S3, The Raman peaks corresponding to the monoclinic phase of VO_2 gradually weaken and broaden as the temperature approaches the critical temperature. No new Raman modes associated with other phases (such as V_2O_5 or V_2O_3) appear during the heating process. The broadening and eventual disappearance of Raman peaks are consistent with the first-order phase transition of VO_2, without evidence of secondary phase formation or irreversible lattice damage. These results indicate that the modulation effects observed in the main manuscript are primarily due to reversible electronic changes intrinsic to the VO_2 MIT.

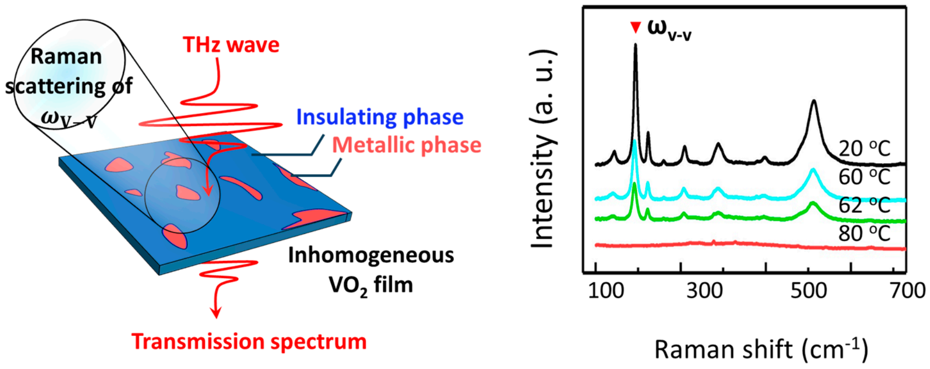

Figure S3. Temperature-dependent Raman spectra of the epitaxial VO_2 film during the MIT process. Adapted from Lu, C., et al., *J. Appl. Phys.*, **135**, 2024, DOI: 10.1063/5.0209629, licensed under CC BY-NC-ND 4.0.

Section S4. THz properties of VO_2 film by Drude-Smith model

It is generally regarded that VO_2 undergoes an inhomogenous MIT due to its first-order phase transition, where metallic (rutile) phase and insulating (monoclinic) phase debate at crystallite impurities. Setting the possibility of a VO_2 nanodomain transformed from insulating state into metallic state at temperature T obeys Gaussian distribution $N(T_c, \Delta T^2)$, where T_c is the temperature with the most phase-transition possibility, and $2\Delta T$ reflects the temperature window of the MIT. Thus, the metallic phase volume fraction p_m can be modeled by integrating a Gaussian distribution function:

$$p_m(T) = \int_0^T \frac{1}{\sqrt{2\pi}\Delta T} \exp \frac{-(T-T_c)^2}{2\Delta T^2} dT \qquad \text{(S4.1)}$$

where $T_c = 60.4°\mathrm{C}$ and $\Delta T = 3.8\,°\mathrm{C}$ are acquired from in-situ Raman spectroscopy measurements in previous research (Lu, C., et al., *J. Appl. Phys.*, 135, 2024).

Whereas metallic phase volume fraction p_m reflects the phase transition from macroscopic perspective, THz properties influenced by microscopic carrier dynamics could be modelled by the Drude-Smith model:

$$\tilde{\sigma}(\omega) = \frac{Ne^2\tau/m*}{1-i\omega\tau}\left(1 + \frac{c_1}{1-i\omega\tau}\right) \qquad \text{(S4.2)}$$

where $\tilde{\sigma} = \sigma_1 + i\sigma_2$ is the complex electrical conductivity, N is the carrier density, e is the elementary charge, m^* is the effective mass of charge carriers, τ is the scattering time, and c_1 is the confinement factor. Precisely, we set $m* = 23m_e$, $\tau = 20\,fs$ according to previous researches, N and c_1 are functions of temperature:

$$N = p_m(T) \times N_0 \qquad \text{(S4.3a)}$$

$$c_1 = A2 + (A1 - A2)/\Big(1 + exp\big((T - T^*)/dT\big)\Big) \qquad \text{(S4.3b)}$$

where N_0 is the carrier density at fully metallic state, and Eq. (S4.3b) is a Boltzmann function with parameters in need of correction. The detailed c_1 and N_0 can be extracted from DC conductivity, obeying

$$\sigma_{DC} = \tilde{\sigma}(0) = Ne^2\tau/m*(1 + c_1) \qquad \text{(S4.4)}$$

The as-corrected c_1 and N_0 are plotted in Figure S4 (a) and (b). The parameters in Eq. (S4.3b) are fitted to $A1 = -1$, $A2 = -0.75$, $T^* =$

64°C, and dT=1.23°C. N_0 in Eq. (S4.3a) is fitted to 5×10^{22} cm^{-3}. The resulted $\tilde{\sigma}(0)$ fit the measured DC conductivity well in Figure S4 (c).

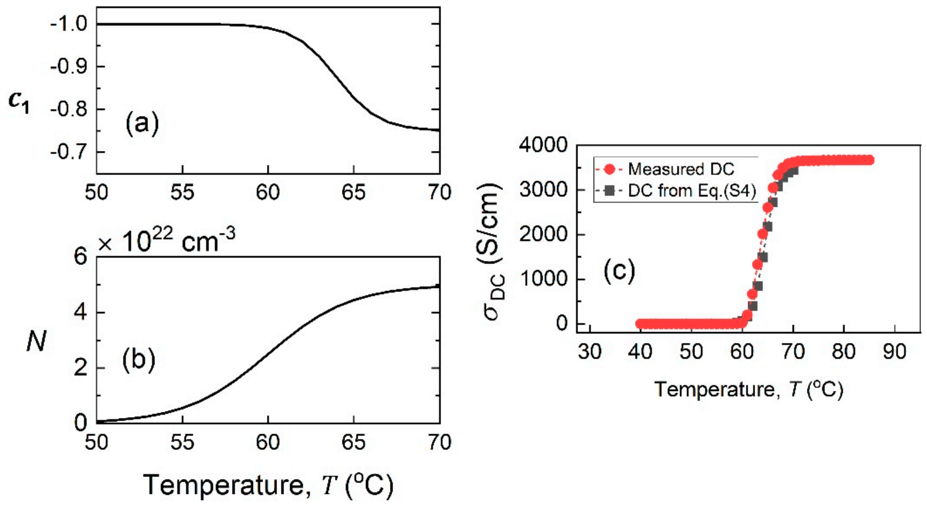

Figure S4. The as-corrected c_1 and N_0 are plotted in Figure S4 (a) and (b). (c) The resulting $\tilde{\sigma}(0)$ as a function of c_1 and N_0, obeying Eq. (S4.4).

Section S5. Experiment setup

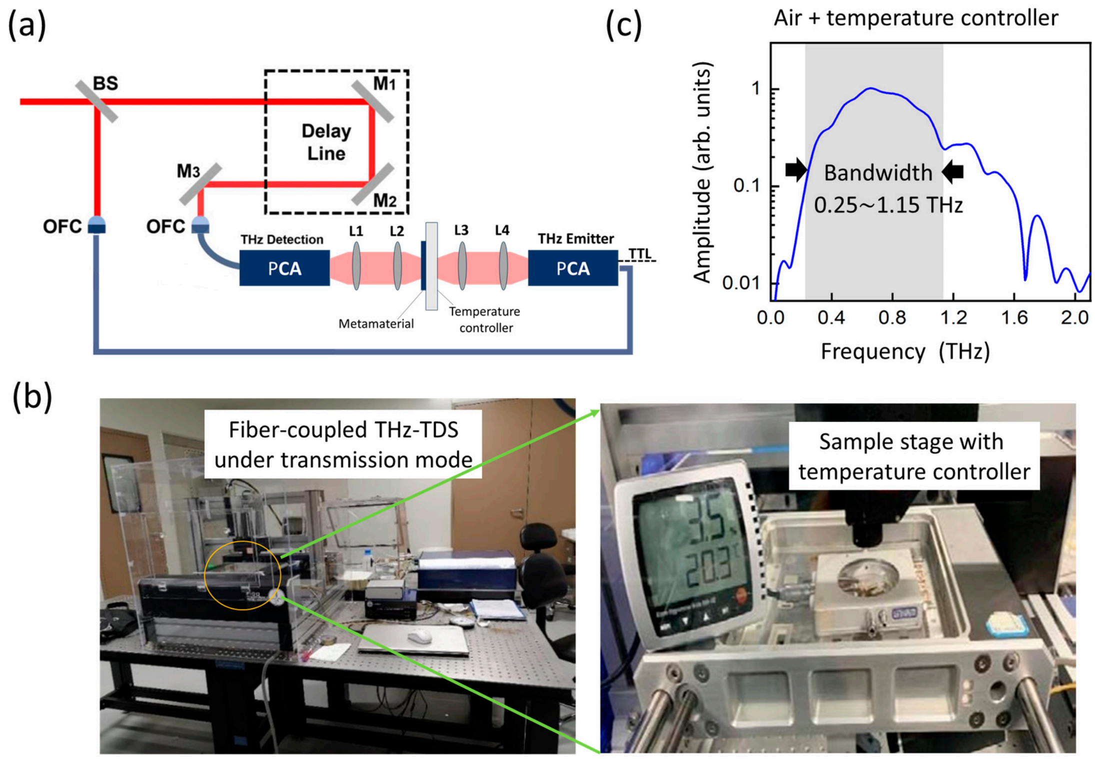

Figure S5. Schematic (a) and practical experiment setup (b) of THz-TDS under transmission mode, with a temperature controller to trigger the MIT of VO_2-hybrid metamaterial. (c) Transmission spectra of this set up, revealing bandwidth at around 0.25~1.15 THz.

Section S6. Spectra in the cooling process & cycling stability

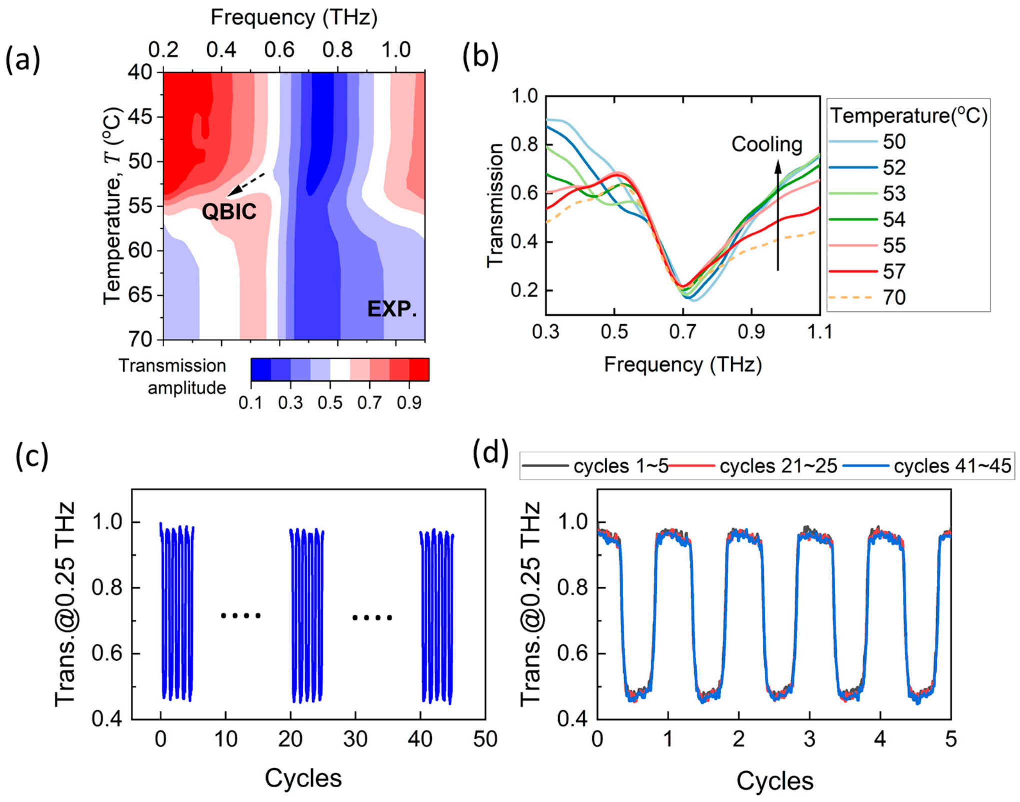

Figure S6. (a) Transmission amplitude in the cooing process as a function of temperature and frequency, with QBIC frequency traced by black arrow. (b) transmission spectra at representative temperatures.

Figure S6. (c) The experiment demonstrates the cycling stability of the qBIC metamaterial. The material undergoes heating and cooling cycles between 40°C and 70°C at a rate of 20°C/min. Transmission at 0.25 THz for cycles 1–5, 21–25, and 41–45 are analyzed.

(d) A comparison of the transmission at cycles 1–5, 21–25, and 41–45 shows no significant change in modulation depth, revealing the thermal stability of the metamaterial within 45 cycles.
